# The Relationship Between Prediabetes and Bone Mass in Adolescents: Analysis of the National Health and Nutrition Examination Survey From 2005 to 2010

**DOI:** 10.3389/fendo.2021.749998

**Published:** 2021-10-25

**Authors:** Chun-Ming Ma, Fu-Zai Yin

**Affiliations:** Department of Endocrinology, The First Hospital of Qinhuangdao, Qinhuangdao, China

**Keywords:** prediabetes, impaired fasting glucose, impaired glucose tolerance, bone mineral density, adolescents

## Abstract

**Objective:**

The purpose of this study was to observe the relationship between impaired fasting glucose (IFG), impaired glucose tolerance (IGT), and bone mineral density (BMD) in different sites in adolescents.

**Methods:**

A retrospective study was conducted on adolescents age 12–19 years of the United States. Data were extracted from the National Health and Nutrition Examination Survey (NHANES) 2005–2006, 2007–2008, and 2009–2010 cycles. IFG was defined as fasting plasma glucose (FPG) levels that were ≥5.6 and <7.0 mmol/L. IGT was defined as 2-h plasma glucose levels that were ≥7.8 and <11.1 mmol/L after the oral glucose tolerance test (OGTT).

**Results:**

After controlling for age, gender, race, and body mass index (BMI) *Z*-score, adolescents in different categories of IGT had significantly different levels of areal BMD (aBMD) and bone mineral apparent density (BMAD) (IGT main effect: *P* < 0.05 for all, two-way ANOVA). There was no main effect between different categories of IFG with regard to aBMD and BMAD (*P* > 0.05). There was no interaction between IFG and IGT with regard to aBMD and BMAD (*P* > 0.05). In multiple regression analysis, the 2-h plasma glucose maintained an independent association with femoral neck aBMD (*β *= −0.011, 95% CI: −0.017~−0.006, *P *< 0.001, *R* ^2^ = 0.012), total femur aBMD (*β *= −0.015, 95% CI: −0.021~−0.009, *P *< 0.001, *R* ^2^ = 0.018), total spine aBMD (*β *= −0.015, 95% CI: −0.020~−0.010, *P *< 0.001, *R* ^2^ = 0.018), and total spine BMAD (*β *= −0.002, 95% CI: −0.003~0.000, *P* = 0.006, *R* ^2^ = 0.003).

**Conclusion:**

The present study demonstrates that BMD was decreased in adolescents with IGT. Two-hour plasma glucose, not FPG, negatively correlated with BMD. The effect of 2-h plasma glucose was consistent across the sites of bone.

## Introduction

Osteoporosis is a common public health problem that also imposes a tremendous burden. Diabetes is another major public health problem globally. A large number of studies have confirmed the effect of diabetes on bone metabolism. Bone mineral density (BMD) is decreased in type 1 diabetes but increased in T2DM, compared with controls. Both type 1 diabetes and type 2 diabetes are associated with decreased bone strength as well as increased fracture risk (1).

Prediabetes is an in-between condition where blood glucose levels are higher than normal, but not high enough yet to be diagnosed as diabetes. Prediabetes also impact bone metabolism. Bone turnover was lower in those with prediabetes (2). Trabecular bone score, an index of bone quality, was lower in prediabetes. Prediabetes was associated with deterioration of bone microarchitecture (3, 4). Chen et al. analyzed the data from the United States National Health and Nutrition Examination Surveys (NHANES) during the period from 2005 to 2014. The results showed that populations with prediabetes may be exposed to relatively higher BMD but a higher prevalence of fracture (5). In postmenopausal women, osteoporosis was more common in the prediabetes group than in the control group (6). A nationwide population-based cohort study of the Republic of Korea demonstrated that the risks of hip fractures started to increase in prediabetes (7).

Bone is a living tissue that changes throughout our lives. Childhood and adolescence are the most important time for building a strong skeleton. In children and adolescents, the studies about glucose metabolism and bone are focused on T1DM patients. T1DM plays a role in decreasing BMD *Z*-scores in the whole body and lumbar spine in children and adolescents (8). Hyperglycemia and insulin deficiency can affect bone cell functions, as well as the bone marrow fat, thus impairing the bone strength, geometry, and microarchitecture (9). With the epidemic of childhood obesity, the prevalence of prediabetes among children and adolescents has increased over the past decade (10). Unlike adults, Pollock et al. found that total body bone mineral content (BMC) was 4% lower in overweight children with prediabetes than in those without prediabetes (11). Conversely, Afghani et al. found that the levels of BMC or BMD between children with impaired glucose tolerance (IGT) or normal glucose tolerance were similar in overweight Latino children (12). The different results may be due to the different age, race, or definition of prediabetes.

However, some questions remain unclear in children and adolescents. First, the age ranges were 7 to 11 and 8 to 13 years in these two studies, respectively. There was no study about 14- to 19-year age ranges. Second, as we know, prediabetes includes impaired fasting glucose (IFG) and IGT. No study compared the effect of IFG and IGT on bone metabolism. Third, is the effect of prediabetes consistent across the sites of bone?

The NHANES is a survey research program conducted by the National Center for Health Statistics (NCHS) to assess the health and nutritional status of adults and children in the United States. The NHANES is conducted continuously and has a large sample size. We can obtain the results about the oral glucose tolerance test (OGTT) and femur and spine BMD in adolescents aged 12 to 19 years from NHANES. In our study, we analyzed the relationship between IFG, IGT, and BMD in different sites in adolescents by using the NHANES database.

## Methods

### Study Design

This cross-sectional study analyzed data from the NHANES. All NHANES data collection protocols were approved by the National Center of Health Statistics Research Ethics Review Board. From 2005 to 2016, the survey started including measurements of OGTT. The femur and spine BMD was not measured in 2011–2012 and 2015–2016 cycles and in subjects with age <40 years in the 2013–2014 cycle. Hence, only three cycles (2005–2006, 2007–2008, and 2009–2010) of data were included in the analysis for this study. This study included adolescents aged ≤19 years. The exclusion criteria were as follows: 1) data about femur and spine BMD missing, 2) data about OGTT missing, 3) subjects have previous medical history of diabetes or taking diabetic pills or insulin to lower blood sugar, 4) subjects had fasting plasma glucose (FPG) levels that were ≥7.0 mmol/L or 2-h plasma glucose levels that were ≥11.1 mmol/L after OGTT, and 5) data about covariates missing. Ethical approval was not required for this study because the study was based on secondary analyses of publicly available data.

### Bone Mineral Density

The femur scans and spine scans were acquired with Hologic QDR-4500A fan-beam densitometers (Hologic, Inc., Bedford, MA, USA) and software version Discovery v12.4 in 2005–2006 through 2009–2010. Whole body scans were taken with Hologic QDR-4500A fan-beam densitometer (Hologic, Inc., Bedford, MA, USA) and Hologic software version 8.26:a3* in 2005–2006. Measurements include bone mineral content (BMC) (g) and bone area (cm^2^). Areal bone mineral density (aBMD) (g/cm^2^) was calculated as follows: aBMD (g/cm^2^) = BMC (g)/bone area (cm^2^). Total spine BMD included L1–L4 vertebra BMD. Total spine bone mineral apparent density (BMAD, g/cm^3^) was also calculated as follows: Total spine BMAD (g/cm^3^) = (BMC_1_ + BMC_2_ + BMC_3_ + BMC_4_)/(*V*_1_ + *V*_2_ + *V*_3_ + *V*_4_), where BMC*_n_* is the BMC of the *n*th vertebrae, and *V_n_* is the volume of the *n*th individual vertebra = bone area*_n_* ^1.5^ (13, 14).

Longitudinal monitoring was conducted through the daily spine phantom scans as required by the manufacturer and the once-weekly femur phantom scans in order to correct any scanner-related changes in participant data. The circulating HSP-Q96 and block phantoms, which were scanned at the start of operations at each site, provided additional data for use in longitudinal monitoring and cross calibration.

### OGTT Test

Beginning in 2005, an OGTT was added to the laboratory protocol. A fasting glucose blood test was performed on all participants, 12 years and older, who were examined in the morning session, after a 9-h fast. After the initial venipuncture, participants were asked to drink a calibrated dose (generally 75 g of glucose) of Trutol and had a second venipuncture 2 h (plus or minus 15 min) after drinking the Trutol. Glucose concentration was determined by a hexokinase method. Insulin concentration was determined by the Merocodia Insulin ELISA. The following equation was used to calculate the homeostasis model assessment insulin resistance (HOMA-IR) index: (fasting insulin level × fasting glucose level)/22.5 (15).

### Definition of the Types of Prediabetes

IFG was defined as FPG levels that were ≥5.6 and <7.0 mmol/L. IGT was defined as 2-h plasma glucose levels that were ≥7.8 and <11.1 mmol/L after OGTT (16). These subjects were divided into four groups according to the levels of glucose: 1) normal glucose regulation group (NGR group): normal FPG and 2-h plasma glucose; 2) IFG group: IFG and normal 2-h plasma glucose; 3) IGT group: normal FPG and IGT; and 4) impaired glucose regulation group (IGR group): both IFG and IGT.

### Covariates

Sociodemographic covariates included age, gender, and ethnicity (white *vs*. non-white). Body measures included body mass index (BMI) and waist circumference (WC). Laboratory measures included triglyceride (TG), C-reactive protein (CRP), and 25-hydroxyvitamin D [25(OH)D].

In the 2005–2006 cycle, total 25(OH)D (sum of 25-hydroxyvitamin D2 and 25-hydroxyvitamin D3) was measured by a radioimmunoassay (DiaSorin) method. In the 2007–2010 cycles, 25(OH)D was measured by an ultra-high-performance liquid chromatography tandem mass spectrometric (LC-MS/MS) method. The following regression equation was used to convert RIA to LC-MS/MS-equivalents for NHANES 2005–2006: LC-MS/MS_equivalent_ = 8.36753 + 0.97012 * RIA_original_.

*Z*-score and percentile for BMI were calculated according to CDC curves (https://www.cdc.gov/growthcharts/percentile_data_files.htm). The data were stratified to underweight (<5th percentile), healthy weight (5th–84th percentile), overweight (85th–94th percentile), and obesity (≥95th percentile) based on BMI *Z*-score *(* 17).

### Statistical Analyses

Analyses were performed using the SPSS 24.0 statistical software (SPSS 24.0 for Windows; SPSS, Inc., Chicago, IL, USA) and STATA version 16.0 software (STATA Corporation, TX, USA). Quantitative data were expressed as mean with standard deviation. When not normally distributed, the data were ln-transformed for analysis and are expressed as medians with interquartile ranges. The four groups were compared using the analysis of variance (ANOVA). Categorical data were compared by using the chi-square test. We also ascertained age, gender, race, and BMI *Z*-score adjusted means of BMD across different phenotypes of prediabetes by using general linear models. Significant differences across different phenotypes of prediabetes were searched by using two-factor ANOVA (normal FPG *vs*. IFG and normal 2-h plasma glucose *vs*. IGT), and the main effects of IFG and IGT and the IFG × IGT interaction were tested. A Pearson correlation coefficient was used to measure the strength of association between variables. Multiple linear regression analyses were performed to examine the relationships between BMD, glucose, and other variables. WC was not used because of collinearity with BMI. The analysis was also stratified by BMI category. *P <*0.05 was considered statistically significant.

## Results

**Figure 1:**
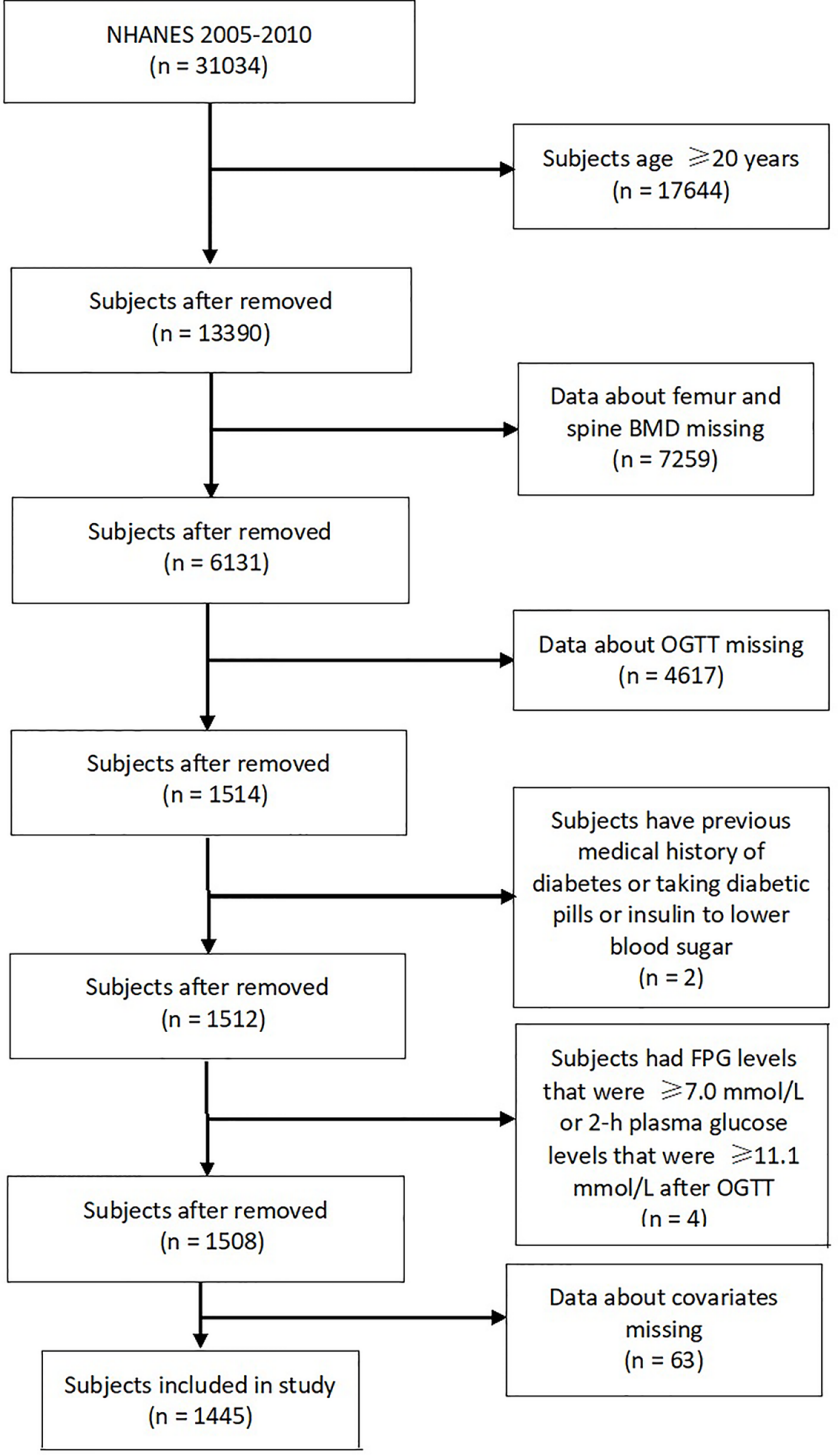
Flowchart of the selection of participants.

**Figure 1** summarizes the selection process of the study. Eventually, a total of 1,445 subjects (807 boys and 638 girls), aged 15.9 ± 2.3 years were enrolled in this study. Of these subjects, 230 (15.9%) had IFG only, 46 (3.2%) had IGT only, and 20 (1.4%) had both IFG and IGT.

Demographic information, as well as anthropometric, biochemical, and BMD data, are presented in **Table 1**. The levels of age were lower in subjects in the IGT group than subjects in the NGR group and IFG group (*P *< 0.05). The frequencies of boys were higher in subjects in the IFG group than subjects in the NGR, IGT, and IGR groups (*P *< 0.05). The proportions of race were similar in the four groups (*P* > 0.05). BMI and WC were all significantly higher in the IGR than in the NGR, IFG, and IGT groups (*P *< 0.05). BMI *Z*-score was significantly higher in the IFG and IGR groups than in the NGR (*P *< 0.05). The levels of TG were higher in subjects in the IFG, IGT, and IGR groups than subjects in the NGR group (*P *< 0.05). The levels of CRP were higher in subjects in the IGT and IGR groups than subjects in the NGR group (*P *< 0.05). 25(OH)D was significantly lower in the IGR than in the NGR, IFG, and IGT groups (*P *< 0.05). The levels of HOMA-IR were higher in subjects in the IFG, IGT, and IGR groups than subjects in the NGR group and were higher in subjects in the IGR group than subjects in the IFG and IGT groups (*P *< 0.05). The levels of BMC were lower in subjects in the IGT group than subjects in the NGR and IFG groups (*P *< 0.05). The levels of femoral neck aBMD and total femur aBMD were lower in subjects in the IGT group than subjects in the NGR, IFG, and IGR groups (*P *< 0.05). The levels of total spine aBMD were lower in subjects in the IFG and IGT groups than subjects in the NGR group (*P *< 0.05). The levels of total spine BMAD were lower in subjects in the IFG group than subjects in the NGR group (*P *< 0.05).

**Table 1:** Clinical and laboratory characteristics of the subjects in each group.

Variable	NGR group (*n* = 1,149)	IFG group (*n* = 230)	IGT group (*n* = 46)	IGR group (*n* = 20)	*F* or *χ* ^2^	*P*
Age (years), mean (SD)	16.0 (2.3)	15.9 (2.4)	14.8 (2.2)^a^ ^,^ ^b^	15.9 (2.3)	3.856	0.009
Gender [boy (%)]	600 (52.2)	174 (75.7)^a^	23 (50.0)^b^	10 (50.0)^b^	43.634	<0.001
Race [White (%)]	328 (28.5)	63 (27.4)	14 (30.4)	4 (20.0)	0.910	0.823
Height (cm), mean (SD)	165.7 (10.1)	167.9 (10.3)^a^	160.5 (8.7)^a^ ^,^ ^b^	166.7 (10.8)^c^	7.633	<0.001
Weight (kg), mean (SD)	65.3 (17.8)	69.4 (20.1)^a^	63.5 (18.1)^b^	79.4 (25.1)^a^ ^,^ ^b^ ^,^ ^c^	6.994	<0.001
BMI (kg/m^2^), mean (SD)	23.6 (5.5)	24.4 (6.1)^a^	24.6 (6.6)	28.2 (7.5)^a^ ^,^ ^b^ ^,^ ^c^	5.694	0.001
BMI *Z*-score, mean (SD)	0.53 (1.12)	0.69 (1.16)^a^	0.82 (1.14)	1.30 (1.33)^a^ ^,^ ^b^	4.842	0.002
WC (cm), mean (SD)	80.9 (13.7)	84.7 (15.8)^a^	85.1 (16.4)^a^	92.6 (17.2)^a^ ^,^ ^b^ ^,^ ^c^	9.541	<0.001
TG (mmol/L), median (IQR)	0.77 (0.56~1.07)	0.93 (0.65~1.33)^a^	0.99 (0.77~1.34)^a^	1.34 (0.97~1.76)^a^ ^,^ ^b^	16.860	<0.001
CRP (mg/dl), median (IQR)	0.04 (0.02~0.13)	0.05 (0.02~0.16)	0.07 (0.02~0.19)^a^	0.17 (0.04~0.41)^a^ ^,^ ^b^	5.063	0.002
25(OH)D (nmol/L), mean (SD)	58.6 (20.6)	60.2 (20.5)	60.9 (19.7)	48.3 (17.1)^a^ ^,^ ^b^ ^,^ ^c^	2.293	0.076
FPG (mmol/L), mean (SD)	5.09 (0.30)	5.82 (0.20)^a^	5.12 (0.31)^b^	5.93 (0.20)^a^ ^,^ ^c^	473.159	<0.001
OGTT, 2-h plasma glucose (mmol/L), mean (SD)	5.13 (1.02)	5.73 (1.06)^a^	8.53 (0.81)^a^ ^,^ ^b^	8.47 (0.76)^a^ ^,^ ^b^	241.522	<0.001
Fasting insulin (μIU/ml), median (IQR)	10.0 (7.1~14.9)	13.5 (9.7~21.3)^a^	15.8 (9.0~25.1)^a^	26.3 (18.5~37.2)^a^ ^,^ ^b^ ^,^ ^c^	34.535	<0.001
HOMA-IR, median (IQR)	2.25 (1.61~3.39)	3.50 (2.48~5.54)^a^	3.66 (2.08~5.93)^a^	6.95 (4.94~10.13)^a^ ^,^ ^b^ ^,^ ^c^	55.428	<0.001
Total body BMC (g), mean (SD)^d^	2,170.6 (523.8)	2,193.6 (522.2)	1,894.5 (393.0)^a^ ^,^ ^b^	1,984.4 (329.3)	2.675	0.046
Total body aBMD (g/cm^2^), mean (SD)^d^	1.10 (0.13)	1.08 (0.13)	1.02 (0.11)^a^	1.02 (0.12)	4.097	0.007
Femoral neck BMC (g), mean (SD)	4.57 (1.04)	4.75 (1.03)^a^	3.96 (0.78)^a^ ^,^ ^b^	4.64 (1.46)^c^	7.521	<0.001
Femoral neck aBMD (g/cm^2^), mean (SD)	0.92 (0.16)	0.92 (0.15)	0.84 (0.14)^a^ ^,^ ^b^	0.92 (0.21)^c^	3.897	0.009
Total femur BMC (g), mean (SD)	33.93 (9.60)	35.99 (9.54)^a^	27.93 (7.13)^a^ ^,^ ^b^	32.77 (11.68)	9.643	<0.001
Total femur aBMD (g/cm^2^), mean (SD)	0.99 (0.17)	0.99 (0.16)	0.89 (0.14)^a^ ^,^ ^b^	0.99 (0.19)^c^	5.763	0.001
Total spine BMC (g), mean (SD)	55.18 (14.41)	53.72 (14.83)	44.93 (12.54)^a^ ^,^ ^b^	50.69 (15.01)	8.278	<0.001
Total spine aBMD (g/cm^2^), mean (SD)	0.96 (0.16)	0.92 (0.16)^a^	0.87 (0.16)^a^ ^,^ ^b^	0.91 (0.16)	7.063	<0.001
Total spine BMAD (g/cm^3^), mean (SD)	0.25 (0.04)	0.24 (0.03)^a^	0.24 (0.04)	0.25 (0.03)	5.116	0.002

Values were expressed as mean (SD), and when not normally distributed, they were ln-transformed for analysis and were expressed as medians (IQR).

a Compared with the NGR group, P < 0.05.

b Compared with the IFG group, P < 0.05.

c Compared with the IGT group, P < 0.05.

d In these subjects, 691 subjects have whole body scans; NGR group, n = 563; IFG group, n = 97; IGT group, n = 26; and IGR group, n = 5.

SD, standard deviation; IQR, interquartile range; NGR, normal glucose regulation; IFG, impaired fasting glucose; IGT, impaired glucose tolerance; IGR, impaired glucose regulation; BMI, body mass index; WC, waist circumference; TG, triglyceride; CRP, C-reactive protein; 25(OH)D, 25-hydroxyvitamin D; FPG, fasting plasma glucose; OGTT, oral glucose tolerance test; HOMA-IR, homeostasis model assessment insulin resistance; BMC, bone mineral content; aBMD, areal bone mineral density; BMAD, bone mineral apparent density.

Multivariate-adjusted means of BMD across different phenotypes of prediabetes are given in **Table 2**. After controlling for age, gender, race, and BMI *Z*-score, adolescents in different categories of IGT had significantly different levels of aBMD and BMAD (IGT main effect: *P* < 0.05 for all, two-way ANOVA). After controlling for age, gender, race, and BMI *Z*-score, adolescents with IGT have lower levels of aBMD and BMAD than adolescents with normal 2-h plasma glucose. There was no main effect between different categories of IFG with regard to aBMD and BMAD (*P* > 0.05). There was no interaction between IFG and IGT with regard to aBMD and BMAD (*P* > 0.05).

**Table 2:** Multivariate-adjusted means for bone mineral density across phenotypes of prediabetes.

BMD	Normal OGTT, 2-h plasma glucose	IGT^a^	*P* ^b^		
			IFG	IGT	IFG*IGT
Total body aBMD (g/cm^2^)^c^					
Normal FPG	1.102 ± 0.004	1.040 ± 0.020	0.169	0.009	0.881
IFG^a^	1.071 ± 0.010	1.002 ± 0.045			
Femoral neck aBMD (g/cm^2^)					
Normal FPG	0.920 ± 0.004	0.856 ± 0.019	0.847	0.015	0.214
IFG^a^	0.902 ± 0.008	0.881 ± 0.028			
Total femur aBMD (g/cm^2^)					
Normal FPG	0.998 ± 0.004	0.917 ± 0.019	0.699	0.004	0.099
IFG	0.975 ± 0.009	0.953 ± 0.029			
Total spine aBMD (g/cm^2^)					
Normal FPG	0.954 ± 0.003	0.897 ± 0.018	0.176	0.001	0.979
IFG	0.932 ± 0.008	0.875 ± 0.027			
Total spine BMAD (g/cm^3^)					
Normal FPG	0.251 ± 0.001	0.245 ± 0.004	0.050	0.012	0.355
IFG	0.247 ± 0.002	0.234 ± 0.006			

Mean ± standard error (all such values).

a IFG: FPG ≥5.6 and <7.0 mmol/L; IGT: OGTT, 2-h plasma glucose ≥7.8 and <11.1 mmol/L. Normal FPG and OGTT, 2-h plasma glucose, n = 1,149; normal FPG and IGT, n = 46; IFG and normal OGTT, 2-h plasma glucose, n = 230; IFG and IGT, n = 20.

b ANOVA, adjusted for age, gender, race, and BMI Z-score.

c In these subjects, 691 subjects have total body aBMD. Normal FPG and OGTT, 2-h plasma glucose, n = 563; normal FPG and IGT, n = 97; IFG and normal OGTT, 2-h plasma glucose, n = 26; IFG and IGT, n = 5.

aBMD, areal bone mineral density; BMAD, bone mineral apparent density; OGTT, oral glucose tolerance test; FPG, fasting plasma glucose; IFG, impaired fasting glucose; IGT, impaired glucose tolerance; BMI, body mass index.

The correlation coefficients between aBMD and BMAD and glucose for all of the subjects are shown in **Table 3**. FPG was negatively correlated with total spine aBMD (*r *= −0.077, *P* = 0.003) and total spine BMAD (*r *= −0.104, *P* = 0.003). Two-h plasma glucose was negatively correlated with femoral neck aBMD (*r *= −0.101, *P *< 0.001), total femur aBMD (*r *= −0.140, *P *< 0.001), total spine aBMD (*r *= −0.159, *P *< 0.001), and total spine BMAD (*r *= −0.060, *P* = 0.022). HOMA-IR was positively correlated with femoral neck aBMD (*r* = 0.088, *P* = 0.001), total femur aBMD (*r* = 0.058, *P* = 0.027), total spine aBMD (*r* = 0.060, *P* = 0.023), and total spine BMAD (*r* = 0.137, *P *< 0.001).

**Table 3:** Simple correlations between bone mineral density and glucose in adolescents.

Variable	Femoral neck BMC		Femoral neck aBMD		Total femur BMC		Total femur aBMD		Total spine BMC		Total spine aBMD		Total spine BMAD	
	*r*	*P*	*r*	*P*	*r*	*P*	*r*	*P*	*r*	*P*	*r*	*P*	*r*	*P*
All (*n* = 1,445)														
FPG (mmol/L)	0.100	<0.001	0.046	0.082	0.105	<0.001	0.035	0.185	−0.025	0.333	−0.077	0.003	−0.104	<0.001
OGTT, 2-h plasma glucose (mmol/L)	−0.133	<0.001	−0.101	<0.001	−0.183	<0.001	−0.140	<0.001	−0.234	<0.001	−0.159	<0.001	−0.060	0.022
Fasting insulin (μIU/ml)	0.059	0.025	0.086	0.001	−0.007	0.792	0.056	0.033	−0.041	0.116	0.072	0.006	0.156	<0.001
HOMA-IR	0.068	0.009	0.088	0.001	0.006	0.826	0.058	0.027	−0.043	0.104	0.060	0.023	0.137	<0.001
Underweight (*n* = 42)														
FPG (mmol/L)	0.125	0.432	0.206	0.190	0.075	0.638	0.110	0.487	−0.013	0.936	−0.092	0.562	−0.155	0.328
OGTT, 2-h plasma glucose (mmol/L)	−0.233	0.137	−0.183	0.246	−0.230	0.142	−0.117	0.462	−0.088	0.581	−0.051	0.748	−0.006	0.970
Fasting insulin (μIU/ml)	−0.181	0.251	−0.146	0.357	−0.131	0.409	−0.072	0.650	−0.188	0.234	−0.155	0.327	−0.084	0.599
HOMA-IR	−0.154	0.329	−0.108	0.497	−0.114	0.474	−0.053	0.739	−0.181	0.250	−0.162	0.306	−0.102	0.520
Healthy weight (*n* = 871)														
FPG (mmol/L)	0.064	0.059	0.005	0.884	0.094	0.005	0.006	0.848	−0.039	0.251	−0.125	<0.001	−0.178	<0.001
OGTT, 2-h plasma glucose (mmol/L)	−0.216	<0.001	−0.190	<0.001	−0.236	<0.001	−0.211	<0.001	−0.277	<0.001	−0.231	<0.001	−0.140	<0.001
Fasting insulin (μIU/ml)	−0.114	0.001	−0.087	0.011	−0.122	<0.001	−0.095	0.005	−0.090	0.008	−0.027	0.433	0.035	0.305
HOMA-IR	−0.099	0.003	−0.082	0.016	−0.102	0.002	−0.089	0.008	−0.092	0.007	−0.044	0.196	0.007	0.839
Overweight (*n* = 255)														
FPG (mmol/L)	0.085	0.178	0.016	0.805	0.077	0.220	−0.030	0.638	−0.061	0.331	−0.089	0.156	−0.088	0.163
OGTT, 2-h plasma glucose (mmol/L)	−0.214	0.001	−0.205	0.001	−0.232	<0.001	−0.269	<0.001	−0.313	<0.001	−0.279	<0.001	−0.194	0.002
Fasting insulin (μIU/ml)	−0.270	<0.001	−0.264	<0.001	−0.269	<0.001	−0.279	<0.001	−0.248	<0.001	−0.183	0.003	−0.091	0.148
HOMA-IR	−0.244	<0.001	−0.248	<0.001	−0.244	<0.001	−0.269	<0.001	−0.243	<0.001	−0.186	0.003	−0.098	0.117
Obesity (*n* = 277)														
FPG (mmol/L)	0.090	0.136	0.014	0.818	0.084	0.164	0.030	0.621	−0.011	0.855	−0.064	0.292	−0.094	0.117
OGTT, 2-h plasma glucose (mmol/L)	−0.207	0.001	−0.193	0.001	−0.246	<0.001	−0.224	<0.001	−0.238	<0.001	−0.197	0.001	−0.111	0.065
Fasting insulin (μIU/ml)	−0.008	0.894	−0.075	0.216	−0.048	0.422	−0.074	0.217	−0.111	0.065	−0.092	0.126	−0.051	0.401
HOMA-IR	0.004	0.946	−0.069	0.251	−0.035	0.561	−0.067	0.267	−0.107	0.076	−0.096	0.109	−0.061	0.309

BMC, bone mineral content; aBMD, areal bone mineral density; BMAD, bone mineral apparent density; FPG, fasting plasma glucose; OGTT, oral glucose tolerance test; HOMA-IR, homeostasis model assessment insulin resistance.

When subjects were stratified by BMI category, 2-h plasma glucose was negatively correlated with femoral neck aBMD, total femur aBMD, and total spine aBMD in subjects with healthy weight, overweight, and obesity (*P *< 0.05). Two-hour plasma glucose was negatively correlated with total spine BMAD in subjects with healthy weight and overweight (*P *< 0.05) (**Table 3**).

When femoral neck aBMD, total femur aBMD, total spine aBMD, and total spine BMAD were considered as the dependent variables in a multiple regression analysis with age, gender, race, BMI *Z*-score, TG, CRP, 25(OH)D, FPG, and 2-h plasma glucose as independent variables (model 1), the 2-h plasma glucose maintained an independent association with femoral neck aBMD (*β *= −0.011, 95% CI: −0.017~−0.006, *P *< 0.001, *R* ^2^ = 0.012), total femur aBMD (*β *= −0.015, 95% CI: −0.021~−0.009, *P *< 0.001, *R* ^2^ = 0.018), total spine aBMD (*β *= −0.015, 95% CI: −0.020~−0.010, *P *< 0.001, *R* ^2^ = 0.018), and total spine BMAD (*β *= −0.002, 95% CI: −0.003~0.000, *P* = 0.006, *R* ^2^ = 0.003) (**Supplementary Table 1**).

When femoral neck aBMD, total femur aBMD, total spine aBMD, and total spine BMAD were considered as the dependent variables in a multiple regression analysis with age, gender, race, BMI *Z*-score, TG, CRP, 25(OH)D, HOMA-IR, and 2-h plasma glucose as independent variables (model 2), the 2-h plasma glucose maintained an independent association with femoral neck aBMD (*β *= −0.010, 95% CI: −0.015~−0.004, *P *< 0.001, *R* ^2^ = 0.012), total femur aBMD (*β *= −0.014, 95% CI: −0.019~−0.008, *P *< 0.001, *R* ^2^ = 0.018), total spine aBMD (*β *= −0.015, 95% CI: −0.020~−0.010, *P *< 0.001, *R* ^2^ = 0.018), and total spine BMAD (*β *= −0.002, 95% CI: −0.003~0.000, *P* = 0.006, *R* ^2^ = 0.003) (**Supplementary Table 2**).

In these subjects, 691 subjects have whole body scans. The levels of total body BMC and aBMD were lower in subjects in the IGT group than subjects in the NGR group (*P *< 0.05) (**Table 1**). After controlling for age, gender, race, and BMI *Z*-score, adolescents with IGT have lower levels of total body aBMD than adolescents with normal 2-h plasma glucose (IGT main effect: *P* < 0.05 for all, two-way ANOVA) (**Table 2**). Two-hour plasma glucose was negatively correlated with total body BMC (*r *= −0.219, *P *< 0.001) and aBMD (*r *= −0.213, *P *< 0.001). When 5total body aBMD was considered as the dependent variable in a multiple regression analysis with age, gender, race, BMI *Z*-score, lean mass, TG, CRP, 25(OH)D, FPG, and 2-h plasma glucose as independent variables (model 1), the 2-h plasma glucose maintained an independent association with total body aBMD (*β *= −0.008, 95% CI: −0.014~−0.003, *P* = 0.003, *R* ^2^ = 0.007) (**Supplementary Table 3**). When total body aBMD was considered as the dependent variable in a multiple regression analysis with age, gender, race, BMI *Z*-score, lean mass, TG, CRP, 25(OH)D, HOMA-IR, and 2-h plasma glucose as independent variables (model 2), the 2-h plasma glucose maintained an independent association with total body aBMD (*β *= −0.006, 95% CI: −0.012~−0.001, *P* = 0.029, *R* ^2^ = 0.004) (**Supplementary Table 3**).

## Discussion

The results of the present study show that BMD levels were decreased in adolescents aged 12~19 years with IGT. The levels of BMD were lower in the IGT group, but not in the IGR group. BMD levels were affected by age, gender, race, and BMI (18, 19). Age, gender, and BMI *Z*-score were unmatched between the four groups. After adjusting for these confounding factor, we found that the means of BMD were lower in both the IGT and IGR groups. In the two-factor ANOVA, IGT, not IFG, had a main effect on BMD in adolescents.

The relationship between 2-h plasma glucose and aBMD has been analyzed in previous research. Jia et al. found that 2-h plasma glucose negatively correlated with femoral neck aBMD and lumbar spine aBMD in women (20). However, the negative correlation was not observed in two studies about children (11, 12). In our study, 2-h plasma glucose negatively correlated with aBMD, and no relationships were observed between FPG and aBMD. Two-hour plasma glucose can explain 1%–2% of the total variance of aBMD in adolescents. A relationship between FPG and aBMD was not found in this study.

Adolescent bone density is closely related to height, and low (or high) bone density relative to that of same-age peers may be attributed to short (or taller) stature (21). The International Society for Clinical Densitometry recommended BMAD as a suitable size adjustment technique (22). Total spine BMAD can eliminate the effect of height on BMD in children and adolescents (23). Some studies found the association between BMAD and risk of fracture in children and adolescents (24, 25). In our study, 2-h plasma glucose also negatively correlated with BMAD.

In previous studies (11, 12), the study populations were overweight. Weight status influenced BMD in children and adolescents. A meta-analysis showed that overweight and obese children have a significantly higher BMD compared with normal weight children (26). In our study, study population was stratified according to BMI *Z*-score. Two-hour plasma glucose negatively correlated with BMD across adolescents with healthy weight, overweight, and obesity. It means that the relationship between 2-h plasma glucose and BMD was independent of weight status.

Femur and spine are both common sites that measure BMD. However, there are some differences between femur BMD and spine BMD. Femoral neck reflects cortical bone and spine reflect trabecular bone. In our study, the effect of IGT is consistent across the sites of bone. Using multivariate regression models, we found that 1.2% of the total variance of femoral neck and 1.8% of the total variance of spine were due to 2-h plasma glucose.

Adolescents with IGT and IGR had higher levels of TG, CRP, and HOMA-IR. We observed an independent association of BMD with TG, CRP, and HOMA-IR levels. Kindler et al. found that HOMA-IR was negatively associated with lumbar spine BMC and total body areal BMD (27). Xiao et al. observed a significantly inverse association between TG level and calcaneus BMD in boys and girls aged 6–16 years in Beijing (28). In adolescent patients, systemic inflammation was also associated with bone health (29, 30). The results show that the presence of lipotoxicity, insulin resistance, and chronic inflammation may lead to a further decline of the BMD in adolescents with IGT.

There are limitations to our study. BMD is influenced by other factors in adolescents, such as age, gender, and pubertal development (31). The study was a secondary analysis. The raw data were downloaded from NHANES. BMD *Z*-score and Tanner stage were not evaluated in NHANES. In addition, the sample of the IGR group is small. It should be validated in a larger population.

In conclusion, the present study demonstrates that BMD was decreased in adolescents with IGT. Two-hour plasma glucose, not FPG, negatively correlated with BMD. The effect of 2-h plasma glucose was consistent across the sites of bone.

## Data Availability Statement

Publicly available datasets were analyzed in this study. These can be found here: https://www.cdc.gov/nchs/nhanes/.

## Author Contributions

C-MM analyzed the data and wrote the paper. F-ZY designed the study. All authors contributed to the article and approved the submitted version.

## Conflict of Interest

The authors declare that the research was conducted in the absence of any commercial or financial relationships that could be construed as a potential conflict of interest.

## Publisher’s Note

All claims expressed in this article are solely those of the authors and do not necessarily represent those of their affiliated organizations, or those of the publisher, the editors and the reviewers. Any product that may be evaluated in this article, or claim that may be made by its manufacturer, is not guaranteed or endorsed by the publisher.
