# Supplementary material for: The Relationship Between Prediabetes and Bone Mass in Adolescents: Analysis of the National Health and Nutrition Examination Survey From 2005 to 2010
Source: Front Endocrinol (Lausanne). 2021 Oct 25;12:749998. doi: 10.3389/fendo.2021.749998 (PMC8572624; doi:10.3389/fendo.2021.749998)
Supplement: Supplementary file 1 [file Table_1.doc]

Supplementary table 1. Multiple linear regression analyses for bone mineral density (Stepwise Method)

| Model 1 | Unstandardized Coefficients B | Std. Error | Standardized Coefficients B | *t* | *P* | 95% *CI* | R^2^ |
| --- | --- | --- | --- | --- | --- | --- | --- |
| Femoral neck aBMD |  |  |  |  |  |  |  |
| (Constant) | 0.521 | 0.035 |  | 14.736 | <0.001 | 0.452~0.591 |  |
| BMI Z score | 0.071 | 0.003 | 0.508 | 21.496 | <0.001 | 0.064~0.077 | 0.162 |
| Age (y) | 0.029 | 0.001 | 0.417 | 19.238 | <0.001 | 0.026~0.032 | 0.333 |
| Gender (1=Boys, 2=Girls) | -0.054 | 0.007 | -0.169 | -8.163 | <0.001 | -0.067~-0.041 | 0.362 |
| OGTT, 2-h Plasma Glucose (mmol/L) | -0.011 | 0.003 | -0.088 | -4.010 | <0.001 | -0.017~-0.006 | 0.374 |
| TG (mmol/L) | -0.027 | 0.007 | -0.087 | -3.980 | <0.001 | -0.041~-0.014 | 0.381 |
| CRP (mg/dL) | -0.010 | 0.003 | -0.084 | -3.570 | <0.001 | -0.015~-0.004 | 0.387 |
| Total femur aBMD |  |  |  |  |  |  |  |
| (Constant) | 0.570 | 0.036 |  | 15.704 | <0.001 | 0.499~0.641 |  |
| Age (y) | 0.032 | 0.002 | 0.450 | 21.214 | <0.001 | 0.029~0.035 | 0.186 |
| BMI Z score | 0.072 | 0.003 | 0.490 | 21.157 | <0.001 | 0.065~0.078 | 0.337 |
| Gender (1=Boys, 2=Girls) | -0.067 | 0.007 | -0.201 | -9.900 | <0.001 | -0.080~-0.054 | 0.379 |
| OGTT, 2-h Plasma Glucose (mmol/L) | -0.015 | 0.003 | -0.113 | -5.246 | <0.001 | -0.021~-0.009 | 0.397 |
| CRP (mg/dL) | -0.012 | 0.003 | -0.099 | -4.312 | <0.001 | -0.017~-0.006 | 0.405 |
| TG (mmol/L) | -0.029 | 0.007 | -0.089 | -4.168 | <0.001 | -0.043~-0.016 | 0.412 |
| Total spine aBMD |  |  |  |  |  |  |  |
| (Constant) | 0.237 | 0.033 |  | 7.162 | <0.001 | 0.172~0.302 |  |
| Age (y) | 0.040 | 0.001 | 0.569 | 28.524 | <0.001 | 0.037~0.042 | 0.298 |
| BMI Z score | 0.059 | 0.003 | 0.416 | 19.137 | <0.001 | 0.053~0.065 | 0.416 |
| Gender (1=Boys, 2=Girls) | 0.060 | 0.006 | 0.185 | 9.708 | <0.001 | 0.048~0.072 | 0.450 |
| OGTT, 2-h Plasma Glucose (mmol/L) | -0.015 | 0.003 | -0.114 | -5.610 | <0.001 | -0.020~-0.010 | 0.468 |
| CRP (mg/dL) | -0.011 | 0.002 | -0.092 | -4.268 | <0.001 | -0.016~-0.006 | 0.475 |
| TG (mmol/L) | -0.026 | 0.006 | -0.083 | -4.108 | <0.001 | -0.039~-0.014 | 0.481 |
| Total spine BMAD |  |  |  |  |  |  |  |
| (Constant) | 0.088 | 0.008 |  | 11.414 | <0.001 | 0.073~0.103 |  |
| Age (y) | 0.007 | 0.000 | 0.442 | 22.275 | <0.001 | 0.007~0.008 | 0.165 |
| Gender (1=Boys, 2=Girls) | 0.029 | 0.001 | 0.389 | 20.460 | <0.001 | 0.027~0.032 | 0.335 |
| BMI Z score | 0.014 | 0.001 | 0.424 | 19.536 | <0.001 | 0.013~0.015 | 0.469 |
| TG (mmol/L) | -0.005 | 0.002 | -0.073 | -3.628 | <0.001 | -0.008~-0.003 | 0.477 |
| CRP (mg/dL) | -0.002 | 0.001 | -0.069 | -3.235 | 0.001 | -0.003~-0.001 | 0.481 |
| OGTT, 2-h Plasma Glucose (mmol/L) | -0.002 | 0.001 | -0.055 | -2.747 | 0.006 | -0.003~0.000 | 0.484 |
| | Race (0=Nonwhite, 1=White) | | --- | | -0.004 | 0.002 | -0.052 | -2.689 | 0.007 | -0.007~-0.001 | 0.486 |

Dependent Variable: bone mineral density. Model 1 BMD was used as the dependent variable in a multiple regression analysis with age, gender, race, BMI Z score, TG, CRP, 25(OH)D, FPG, and OGTT, 2-h plasma glucose as independent variables. TG and CRP were ln-transformed for analysis. BMI: body mass index; TG: triglyceride; CRP: C-reactive protein; 25(OH)D: 25-hydroxyvitamin D; FPG: fasting plasma glucose; OGTT: oral glucose tolerance test; aBMD: areal bone mineral density; BMAD: bone mineral apparent density; BMD: bone mineral density.

Supplementary table 2. Multiple linear regression analyses for bone mineral density (Stepwise Method)

| Model 2 | Unstandardized Coefficients B | Std. Error | Standardized Coefficients B | *t* | *P* | 95% *CI* | R^2^ |
| --- | --- | --- | --- | --- | --- | --- | --- |
| Femoral neck aBMD |  |  |  |  |  |  |  |
| (Constant) | 0.538 | 0.036 |  | 15.066 | <0.001 | 0.468~0.608 |  |
| BMI Z score | 0.076 | 0.004 | 0.543 | 20.700 | <0.001 | 0.069~0.083 | 0.162 |
| Age (y) | 0.028 | 0.001 | 0.410 | 18.878 | <0.001 | 0.025~0.031 | 0.333 |
| Gender (1=Boys, 2=Girls) | -0.053 | 0.007 | -0.166 | -8.006 | <0.001 | -0.066~-0.040 | 0.362 |
| OGTT, 2-h Plasma Glucose (mmol/L) | -0.010 | 0.003 | -0.078 | -3.537 | <0.001 | -0.015~-0.004 | 0.374 |
| HOMA-IR | -0.020 | 0.007 | -0.079 | -3.014 | 0.003 | -0.033~-0.007 | 0.381 |
| CRP (mg/dL) | -0.009 | 0.003 | -0.082 | -3.517 | <0.001 | -0.015~-0.004 | 0.387 |
| TG (mmol/L) | -0.021 | 0.007 | -0.068 | -3.011 | 0.003 | -0.035~-0.007 | 0.391 |
| Total femur aBMD |  |  |  |  |  |  |  |
| (Constant) | 0.588 | 0.037 |  | 16.060 | <0.001 | 0.516~0.660 |  |
| Age (y) | 0.032 | 0.002 | 0.443 | 20.839 | <0.001 | 0.029~0.035 | 0.186 |
| BMI Z score | 0.077 | 0.004 | 0.526 | 20.483 | <0.001 | 0.069~0.084 | 0.337 |
| Gender (1=Boys, 2=Girls) | -0.066 | 0.007 | -0.197 | -9.738 | <0.001 | -0.079~-0.052 | 0.379 |
| OGTT, 2-h Plasma Glucose (mmol/L) | -0.014 | 0.003 | -0.103 | -4.738 | <0.001 | -0.019~-0.008 | 0.397 |
| CRP (mg/dL) | -0.012 | 0.003 | -0.097 | -4.259 | <0.001 | -0.017~-0.006 | 0.405 |
| HOMA-IR | -0.021 | 0.007 | -0.082 | -3.201 | 0.001 | -0.035~-0.008 | 0.412 |
| TG (mmol/L) | -0.023 | 0.007 | -0.070 | -3.143 | 0.002 | -0.037~-0.009 | 0.416 |
| Total spine aBMD |  |  |  |  |  |  |  |
| (Constant) | 0.237 | 0.033 |  | 7.162 | <0.001 | 0.172~0.302 |  |
| Age (y) | 0.040 | 0.001 | 0.569 | 28.524 | <0.001 | 0.037~0.042 | 0.298 |
| BMI Z score | 0.059 | 0.003 | 0.416 | 19.137 | <0.001 | 0.053~0.065 | 0.416 |
| Gender (1=Boys, 2=Girls) | 0.060 | 0.006 | 0.185 | 9.708 | <0.001 | 0.048~0.072 | 0.450 |
| OGTT, 2-h Plasma Glucose (mmol/L) | -0.015 | 0.003 | -0.114 | -5.610 | <0.001 | -0.020~-0.010 | 0.468 |
| CRP (mg/dL) | -0.011 | 0.002 | -0.092 | -4.268 | <0.001 | -0.016~-0.006 | 0.475 |
| TG (mmol/L) | -0.026 | 0.006 | -0.083 | -4.108 | <0.001 | -0.039~-0.014 | 0.481 |
| Total spine BMAD |  |  |  |  |  |  |  |
| (Constant) | 0.088 | 0.008 |  | 11.414 | <0.001 | 0.073~0.103 |  |
| Age (y) | 0.007 | 0.000 | 0.442 | 22.275 | <0.001 | 0.007~0.008 | 0.165 |
| Gender (1=Boys, 2=Girls) | 0.029 | 0.001 | 0.389 | 20.460 | <0.001 | 0.027~0.032 | 0.335 |
| BMI Z score | 0.014 | 0.001 | 0.424 | 19.536 | <0.001 | 0.013~0.015 | 0.469 |
| TG (mmol/L) | -0.005 | 0.002 | -0.073 | -3.628 | <0.001 | -0.008~-0.003 | 0.477 |
| CRP (mg/dL) | -0.002 | 0.001 | -0.069 | -3.235 | 0.001 | -0.003~-0.001 | 0.481 |
| OGTT, 2-h Plasma Glucose (mmol/L) | -0.002 | 0.001 | -0.055 | -2.747 | 0.006 | -0.003~0.000 | 0.484 |
| | Race (0=Nonwhite, 1=White) | | --- | | -0.004 | 0.002 | -0.052 | -2.689 | 0.007 | -0.007~-0.001 | 0.486 |

Dependent Variable: bone mineral density. Model 2 BMD was used as the dependent variable in a multiple regression analysis with age, gender, race, BMI Z score, TG, CRP, 25(OH)D, HOMA-IR, and OGTT, 2-h plasma glucose as independent variables. TG, CRP and HOMA-IR were ln-transformed for analysis. BMI: body mass index; TG: triglyceride; CRP: C-reactive protein; 25(OH)D: 25-hydroxyvitamin D; HOMA-IR: homeostasis model assessment insulin resistance; OGTT: oral glucose tolerance test; aBMD: areal bone mineral density; BMAD: bone mineral apparent density; BMD: bone mineral density.

Supplementary table 3. Multiple linear regression analyses for total body areal bone mineral density (Stepwise Method)

| Model | Unstandardized Coefficients B | Std. Error | Standardized Coefficients B | *t* | *P* | 95% *CI* | R^2^ |
| --- | --- | --- | --- | --- | --- | --- | --- |
| Model 1 |  |  |  |  |  |  |  |
| (Constant) | 0.442 | 0.038 |  | 11.626 | <0.001 | 0.368~0.517 |  |
| Lean mass (kg) | 0.007 | 0.000 | 0.615 | 18.137 | <0.001 | 0.006~0.008 | 0.406 |
| Age (y) | 0.018 | 0.002 | 0.318 | 10.800 | <0.001 | 0.014~0.021 | 0.517 |
| Gender (1=Boys, 2=Girls) | 0.051 | 0.008 | 0.194 | 6.550 | <0.001 | 0.036~0.066 | 0.537 |
| TG (mmol/L) | -0.028 | 0.007 | -0.107 | -4.053 | <0.001 | -0.042~-0.015 | 0.554 |
| OGTT, 2-h Plasma Glucose (mmol/L) | -0.008 | 0.003 | -0.080 | -2.965 | 0.003 | -0.014~-0.003 | 0.561 |
| CRP (mg/dL) | -0.006 | 0.002 | -0.068 | -2.493 | 0.013 | -0.011~-0.001 | 0.565 |
| Model 2 |  |  |  |  |  |  |  |
| (Constant) | 0.451 | 0.038 |  | 11.930 | <0.001 | 0.377~0.525 |  |
| Lean mass (kg) | 0.008 | 0.000 | 0.655 | 18.429 | <0.001 | 0.007~0.009 | 0.406 |
| Age (y) | 0.016 | 0.002 | 0.286 | 9.308 | <0.001 | 0.013~0.019 | 0.517 |
| Gender (1=Boys, 2=Girls) | 0.058 | 0.008 | 0.222 | 7.280 | <0.001 | 0.042~0.074 | 0.537 |
| HOMA-IR | -0.022 | 0.006 | -0.107 | -3.480 | 0.001 | -0.034~-0.009 | 0.560 |
| TG (mmol/L) | -0.020 | 0.007 | -0.076 | -2.735 | 0.006 | -0.035~-0.006 | 0.566 |
| OGTT, 2-h Plasma Glucose (mmol/L) | -0.006 | 0.003 | -0.059 | -2.184 | 0.029 | -0.012~-0.001 | 0.570 |
| CRP (mg/dL) | -0.005 | 0.002 | -0.056 | -2.044 | 0.041 | -0.010~0.000 | 0.573 |

Dependent Variable: Total body areal bone mineral density. Model 1 Total body aBMD was used as the dependent variable in a multiple regression analysis with age, gender, race, BMI Z score, TG, CRP, 25(OH)D, FPG, and OGTT, 2-h plasma glucose as independent variables. Model 2 Total body aBMD was used as the dependent variable in a multiple regression analysis with age, gender, race, BMI Z score, TG, CRP, 25(OH)D, HOMA-IR, and OGTT, 2-h plasma glucose as independent variables. TG, CRP and HOMA-IR were ln-transformed for analysis. BMI: body mass index; TG: triglyceride; CRP: C-reactive protein; 25(OH)D: 25-hydroxyvitamin D; FPG: fasting plasma glucose; HOMA-IR: homeostasis model assessment insulin resistance; OGTT: oral glucose tolerance test; aBMD: areal bone mineral density.
